# The Ethical Aspects of Physical Research

**Published:** 1913-09

**Authors:** De Lacy O'Leary

**Affiliations:** Chairman of Convocation, University of Bristol.


### THE ETHICAL ASPECTS OF PHYSICAL RESEARCH.

BY

The Rev. de Lacy O'Leary, D.D.,

*Chairman of Convocation, University of Bristol.*

---

It is by no means easy to make a satisfactory statement of the teaching of Christian ethics as regards our duty towards animals. In most moral questions we have the guidance of problems considered and worked out in many discussions in the past, but here the usual landmarks are wanting, and we are compelled to make new applications of general principles, a thing which always must be done with the utmost caution. The idea of duty towards what we commonly call the lower animals has only been seriously faced in quite modern times. Many writers, indeed, had laid down definite rules to guide our conduct towards domesticated animals, but these generally expressed a sense of duty towards their owners rather than towards the creatures themselves. In the case of Christian teachers, this omission of a sense of duty towards animals was partly based on the manner in which St. Paul interpreted the precept, "Thou shalt not muzzle the mouth of the ox that treadeth out the corn," on which he remarks, "Doth God take care for oxen ? Or saith He it altogether for our sakes ? For our sakes, no doubt, this is written : that he that ploweth should plow in hope " (1 Cor. ix. 9, 10), whilst scriptural passages in which our Lord spoke of even the sparrows as not forgotten before God were, rightly or wrongly, interpreted rather as bearing upon divine omniscience than upon interest in sparrows.

Nor can we regard this absence of what is now popularly termed "humanitarianism" as at all peculiar to Christianity. Throughout the ancient world we find such a feeling of duty towards animals only in two quarters : in the one where animals were regarded as divine or semi-divine beings, and on this ground treated with superstitious dread and reverence; and in that other which accepted the theory of transmigration. These two exceptions are but apparently so, for in the one the sense of duty was towards the gods, in the other towards fellow-men, the animal form in both merely disguising the real persons. We know that after the collapse of the Persian Empire many Indian and Buddhist theories floated into Europe, chiefly by way of Alexandria, and it appears that such ideas of metempsychosis, which we know prevailed in Pythagorean circles, became known to the Church as one of the phases of gnostic thought, and thus were condemned. The theories of the pre-existence of souls which appear in the Alexandrian Father Origen have a distinctly Oriental tone. Possibly, then, this very dread of gnosticism helped to ward off the Christian thinker from all that seemed to raise animals to the category of persons.

It is perhaps hardly necessary for us to state explicitly that we are considering here only the sense of duty towards animals, and not merely the individual attachment which arises naturally from companionship, or the overflowing love of such an one as St. Francis, which included all God's creation in its embrace.

But at the base of all development of humanitarian feeling lies the fact that it is only the modern advance of psychological thought which has enabled us to regard the animals as sentient beings, and feel that in doing so we have the authority of scientific research.

We may now define the humanitarian theory as an ethical standard which extends the obligation to refrain from causing needless suffering beyond the limits of the human species to such animals as are capable of feeling pain, as we understand the term. Here we are, of course, faced by a very serious difficulty, for we do not know how far we can reasonably regard animals as reflecting our own psychic condition. We can discern many feelings like our own amongst the warm-blooded animals, and apparently an absence of such feelings, or at least a very marked difference, amongst those of the more rudimentary types ; but between these lies a large field, of which our knowledge is very imperfect, nor can we at present venture to draw any clear dividing-line between sentient and non-sentient animals. So far, then, as our own moral duties extend, we must limit the object of our duty to those animals which we reasonably believe to be sentient.

To define these duties more clearly we must premise that in Christian ethics some acts are wrong in the sense that they are definitely forbidden, as we believe, by an expression of the divine will, whilst others are right because commanded by the same authority, whilst a third and larger field contains a number of indeterminate acts which take their colour from the object for which they are performed. In no case is a definitely wrong act permitted for a remoter good, a theory distinctly repudiated by revelation (Romans iii. 8) and by reason, for the immediate object is always more certain than the remoter end in view. We may not steal in order to give the stolen food to a starving person, although the act of taking the food may be ethically justified by our own sincere belief that the food was actually ours, or that the owner consented, or the *bonafide* resolve on our part to compensate the owner for his loss.

Infliction of pain upon man or animal is in itself an act of indeterminate character, taking its colour entirely from the object in view. The surgeon operating on a patient and the murderer slaying his victim may conceivably perform the very same manual act and inflict the same pain, yet the two acts are clearly differentiated by the difference between the object which the operator has in view and that before the murderer. It may even happen that the patient dies, whilst the would-be murderer's victim recovers, yet neither the innocence of the surgeon nor the guilt of the criminal are thereby altered, for in moral theology the latter has the disposition and consequently the sin of murder upon him, even though his victim, contrary to his intention, recover.

Taking, then, the infliction of pain as indeterminate in itself, and deriving its character from the object in view, we have four leading cases before us.

(1) Infliction of pain solely for the purpose of causing pain is obviously in itself evil. Such a desire to cause pain may arise in three chief instances: (*a*) Some persons, it is known, have an abnormal and morbid temperament which finds pleasure in the contemplation of pain. Action on their part merely for the purpose of enjoying the sight of suffering is evil in so far as it is indulged without effort to control, until such a point of mental alienation is reached that the will ceases to control, and so responsibility ceases. To diagnose this is a matter rather for the specialist in mental disease than for ethical inquiry. (*b*) Some have believed that the sight of pain is likely to produce courage and contempt of suffering. Such a theory was held to justify the Roman games, but to the Christian this offers no justification, for the immediate aim in view being to cause pain, this is not justified by a remoter and consequently uncertain object. (*c*) Some may desire to study the phenomena of pain and its expression, but here again the immediate wrong object cannot be excused by the remoter and uncertain aim. In all cases we may safely conclude where the desire is immediately to cause pain we find a definitely evil character.

(2) Contrariwise, where we have no ground or reason for believing that the subject can feel pain, either now or later, there appears no ground for assuming that there is wrong. If we reasonably believe that the subject is of so low an organisation as to be, like the vegetable, incapable of pain, or that it is dead, or so under the influence of an anæsthetic as to be beyond pain, even should it in fact happen that it can actually feel pain contrary to our belief, we are clear from ethical wrong, for it is obvious that there can be no intention to cause pain where we do not believe that pain will be felt.

(3) Where we inflict pain either now or when the animal recovers consciousness, not for the object of causing pain, but for some ulterior object, the act will obviously take its moral colour from that object. Thus the operating surgeon inflicts pain on his patient for the object not of causing pain but of healing some physical defect ; and similarly where the operation is performed upon an aminal for its own good. The ultimate benefit is not certain, but commonly probable. Christian ethics excludes the theory of the rigorist school that no such act is permissible unless the benefit is certain, as it also excludes the lax view that every such act is permissible if there is the vaguest and remotest probability of success. The "probabilior" view that the chances in favour must be more than those adverse, though held by many, seems unsound, because the chances are not really calculable, and in practice this view results in an artificial scrupulosity which is the source of as much harm as good. The " probabilist " view that there need be only some reasonable ground for hoping success would seem the safer and better, in spite of the fact that this view has been fiercely attacked, and is commonly regarded with unmerited disfavour.

(4) Pain may be inflicted for a benefit expected to result, not for the immediate subject, but for the race at large ; it may be for the discovery of remedies which will benefit the human race or sentient animals in general. This is practically the position of vivisection, and here it must be remembered that such research has benefited the animal world as well as the human race. It is not due to the desire to cause pain, for the necessary results can be learned from the unconscious body, so be it that it is discharging those physical functions which do not occur in the dead subject, and thereby allows the operator to learn the effects of drugs, etc., upon the living organism. The only point here where we diverge at all from current humanitarian theory is that we hold it permissible to carry out such research upon the unconscious body of the non-human animal, but not upon the human body even though unconscious. We must remember that there are many forms of research which are possible only where the blood circulates and other corporal functions are at work. Either, then, possible remedies will not be employed with human subjects from lack of previous verification, or will be employed more or less clandestinely by way of experiment. Popular theory tends to condemn research on the ground of our duty towards sentient animals, but we may justly correct this by distinguishing, as seems reasonable, a greater duty towards fellow-members of our own race, and a lesser, though definite, duty towards sentient creatures of the non-human animals. The Christian religion does not seem to authorise us to place men and animals upon precisely the same footing. Again let us repeat that we are not dealing with deliberate torture, which has been considered previously, but with research upon the presently unconscious body. Following our former line of thought, it would appear that the ethical character of the act depends upon whether the operator has before him, in all sincerity, a reasonable ground for hoping success, which will be the case when he puts into operation an experiment which he has already worked out in theory to a favourable conclusion, and which therefore he believes will succeed in practice. It is, of course, fairly open to those who oppose this view to bring forward arguments which tend to reduce the reasonable chance of beneficial result to an improbability, and if thus reduced it will obviously become a wrong.

(5) Again we may suppose the experiment performed not for research but for practice. Thus (*a*) the experimenter reproduces an operation whose conclusions are already well known. Such an experiment, we suppose, can be adequately studied in a text-book, and there is no need to repeat it, its repetition being due only to curiosity. This forms the real charge against vivisection, and there is no doubt that, at a former period, such needless experiments were made by amateurs. This we hold to be wrong, because there is no good end in view, and thus the act, indeterminate in itself, takes its bad colour from the resultant injury to the animal instead of a good colour from an increased knowledge which may benefit both animal and man. The common argument is that this is open to abuse, and thus society becomes responsible for the wrong arising from an act it permits. This is certainly an argument for confining such experiment to members of the medical and veterinary professions, but there we must leave it. Beyond this we can only trust to the conscience and honour of the profession to use this confidence aright, the same conscience and honour on which we rely when we entrust ourselves to the operating surgeon. If we readily give this trust in the greater case, we cannot reasonably withhold it in the lesser. (*b*) Again, experiment may be performed in order that the operator may acquire the manual dexterity necessary for subsequent operations on the human body. It must be a question for the professional surgeon as to whether dexterity and reliability is thereby gained, but to the outsider it would appear that in default of such opportunity, an experiment in vivisection on the human body is performed by every surgeon in his first operation of any particular type.

